# An assessment of the levels of perceived social support among older adults living with HIV and AIDS in Dublin

**DOI:** 10.1186/s40064-016-2302-6

**Published:** 2016-06-16

**Authors:** Nelson Obiora Okonkwo, Fiona Larkan, Marie Galligan

**Affiliations:** Centre for Global Health, University of Dublin Trinity College, 7-9 Leinster Street, Dublin 2, Ireland; Biostatistician, University College Dublin, Belfield, Dublin, Ireland

**Keywords:** HIV, AIDS, Ageing, Intravenous drug use, Social support, Quantitative research

## Abstract

**Objectives:**

To determine the level of perceived social support among older adults living with HIV and AIDS in Dublin.

**Methods:**

The study utilized a cross-sectional survey design to collect data from 46 adults aged 50 years or older who are members of open heart house (OHH) in Ireland, a peer support network for people living with HIV and AIDS. Participants completed a set of interviewer-assisted questionnaires, which included the multidimensional measure of perceived social support (PSS) to assess the level of social support.

**Results:**

Levels of PSS among participants were observed as follows: 54 % had low PSS, 33 % had a moderate PSS and 13 % had high PSS. A fifth of the study participants had a history of injection drug use (IDU) and this group reported higher PSS scores in general than non-IDU’s (*H* = 79.500, Z = −2.678, *p* = 0.006). PSS scores were observed to be higher in general among participants with longer duration of infection [H(2) = 7.856, *p* = 0.020].

**Conclusion:**

Despite its limitations, this study provides vital information about the level of social support among older people living with HIV and AIDS at OHH Dublin. An unexpected but interesting finding was the positive relationship between IDU and PSS level. Formulation of strategies to enable older PLHA to be more proactive members of their community through a peer support network sponsored volunteer services should be encouraged.

## Background

The HIV pandemic is now in its 4th decade since the first case was diagnosed in a homosexual male in 1981. During this time there have been numerous advances, in clinical treatment, with the development of highly active antiretroviral (ARV) medications, and in HIV activism. HIV, a once uniformly fatal infection, has been transformed into a chronic, manageable disease condition (Colvin 2011). HIV can increase morbidity among adults 50 years and older, along with numerous other health issues (such as depression, dementia, insulin resistance, cardiovascular disease and high cholesterol levels) which may arise as a result of ageing (Dau and Holodniy 2008). In fact, HIV and ageing share similar features; they both cause inflammation and suppression of the immune system, can lead to increased depression and loneliness that can result in forgetfulness, non-adherence to medications, an increase in opportunistic infections and death (Leland 2013; Rickabaugh and Jamieson 2010).

According to the World Health Organization report on HIV (Global Health Observatory 2014), 35.0 million people were living with HIV/AIDS worldwide and there were 1.5 million AIDS related deaths in 2013. Globally in 2012, an estimated 3.6 [3.2–3.9] million people aged 50 years and older were living with HIV and AIDS, and in Western Europe and North America this group of older adults makes up 33 % of all adults 15 years and older living with HIV and AIDS (UNAIDS 2013).

The Irish Health Protection and Surveillance Centre (HIV in Ireland 2013) stated that a total of 6979 people have been diagnosed of the disease from the early 1980’s to the end of 2013, and while this figure doesn’t represent the total number of PLWHA in Ireland currently, an estimated 3254 PLWHA accessed HIV outpatient care centres as of the end of 2013. According to the Health Service Executive’s (HSE) Health Protection and Surveillance Centre, 344 new HIV infections were diagnosed in Ireland in 2013, with older adults accounting for 11 %, and an overall prevalence rate of 0.2 % (HIV in Ireland 2013).

## Review of literature

The link between social support and health is that stress and coping mechanisms are aided by support that helps HIV positive individuals adapt to stressful environmental and health challenges (Field and Schuldberg 2011). Numerous studies on older adults living with HIV/AIDS highlight the fact that individuals are at risk of developing poor psychological and physical health if they have inadequate support from friends and family (Field and Schuldberg 2011; Shippy and Karpiak 2005; Heckman et al. 2002). According to research conducted by Shippy and Karpiak (Shippy and Karpiak 2005), individuals over 50 years living with HIV/AIDS are more likely than their HIV negative counterparts to end up in a nursing home, as they will not be able to depend on family members for emotional and financial support. This lack of family support could lead to poor health outcomes in comparison to individuals with a high level of family support. Heckman et al. (2002) carried out research on the psychological symptomatology in this population, using a quantitative self-administered survey of 83 HIV-infected individuals over 50 years of age. Participants with a mean score on the Global Severity Index were compared to values based on 1002 psychiatric outpatients. The results revealed that being white, unemployed and experiencing more HIV symptomatology increases the chances of developing psychological symptoms. Low levels of support from family members and friends, barriers to health due to stigma and ineffectiveness of the mental health system have been associated with increased psychological and social problems (Heckman et al. 2002).

Chesney et al. (2003) carried out research on 199 gay men LWHA who completed a self-report assessment of perceived health functioning, social support and psychological distress. Results of the study showed that social support and health functioning were significantly associated with outcomes of distress and positive affect, and that social support also enhances the overall well-being of older adults more than their younger counterparts. Provision of a sustainable and effective supportive environment to older PLWHA may prove particularly beneficial, suggested by a study conducted in New York City, which reported that older adults aging with HIV have higher unmet needs for support (Schrimshaw and Siegel 2003; Chesney et al. 2003). To support this assertion Shippy and Karpiak (2005) conducted research on the support needs of 160 older adults living with HIV in New York using self-administered questionnaires. Firstly, it was observed that female study participants had larger support networks than male participants which reflected the higher rates of depression observed in males. Secondly, the quality of support that participants received from their networks did not fully address their emotional and material needs; an area of their welfare that has not been fully researched. Results also indicated that physical health status in HIV was a positive indicator of perceived psychosocial and material support (Shippy and Karpiak 2005). On the other hand Lyons et al. (2010) found that in older gay men with HIV/AIDS social support networks correlated with poorer psychological and social well-being, and that younger adults did not fare any better. Social support seeking, spirituality and religiousness and solution centered coping are positive coping mechanisms in this population which in turn can significantly reduce psychological problems in HIV/AIDS patients (Hansen et al. 2013). A study by Emlet and Moceri (2012) identified a different perspective of social support which entails providing emotional and informational supports, and encouraging social connectedness, participation and integration in creating and maintaining elder friendly communities for older adults seeking to keep life satisfaction. The authors further stressed that purposeful and meaningful social interactions in the form of civic engagement and volunteerism systemically delivered through a non-governmental organization such as a church have positive effects on the mental health of this older adult population.

## Rationale

There are currently no specific efforts in place in Ireland to detect the disease in this population nor any public health measures that target this age group in an effort to halt or even reverse the incidence of HIV/AIDS. Older adults living with HIV represent a significant proportion of people living with the disease (11 % of new cases in Ireland) (HIV in Ireland 2013) and this group will require additional medical care and clinical measures as they age with the disease. A recent policy statement suggest that OPLWHA throughout the world continue to be a grossly under researched group in medical, clinical and social science disciplines and that more funding is required to support this research agenda (Emlet 2006). Similarly in other research older adults were generally not perceived to be at high risk for developing HIV even though they had similar HIV risk behaviours as younger adults (Sankar et al. 2011). Factors which were identifiably responsible for such trends include ageism, perceived social support of older adults and their willingness to seek healthcare. The authors further stated that it is important to note that older adults once diagnosed and have commenced treatment score better on quality of life indicators than their younger counterparts a fact that should encourage more in depth analysis and research into the unique socio demographic characteristics of the older PLHA population (Sankar et al. 2011). Hence the purpose of this study is to assess the levels of perceived social support among older adults living with HIV/AIDS in Ireland.

## Objectives

The objectives of this research were to assess the level of perceived social support among older adult members of Open Heart House PLWHA, and also to identify the particular demographic characteristics of the individuals that are associated with lower or higher levels of perceived social support. Social support occurs in many different ways including material/instrumental, emotional and informational supports (Field and Schuldberg 2011; Shippy and Karpiak 2005). But for the purpose of this study social support refers mostly to emotional support derivable from family, friend or a significant other as will be further discussed in the methods section. This study is the first in Ireland on the subject and makes recommendations for future research, particularly in the area of social support needs in adults of increased age.

## Methods

This study utilized a quantitative cross-sectional survey method through administration of a questionnaire instrument between May and August 2013 to obtain information from participants receiving counselling and support services at Open Heart House in Dublin. Open Heart House provides peer support and emotional counselling programmes for people of all age categories, genders and races living in Ireland. The total population of PLWHA who are members at OHH is over 1100 representing about a third of the 3254 PLWHA accessing HIV outpatient care in Ireland; among this OHH population 163 are older PLWHA and comprising of 121 males and 42 females. Eligibility criteria include: adults of 50 years and older, ability to communicate fluently in English and must be a member at Open Heart House Dublin. Study participants were selected from this older population at OHH; the process of recruitment was by non-random convenience sampling of the entire population of HIV positive adults over 50 years who go to OHH for social and emotional supports (Emlet and Moceri 2012). This research method was internationally validated for studying mental health and social support problems in the ageing HIV and AIDS population with a high validity and reliability (Galvan et al. 2008). The survey instrument used for this study was structured in two sections; the first consisted of participant’s bio data and the second contained a set of 12 questions adapted from the multi-dimensional scale of perceived social support (MSPSS) proposed by Zimet, Powell, Farley et al.(Zimet et al. 1990) which elicits individual’s social support from friends and family. This questionnaire assessed the support network from a special person (items 1, 2, 5 and 10), family (items 3, 4, 8 and 11) and friends (items 6, 7, 9, and 12). Responses were collected using a 7-point Likert scale with options ranging from very strongly agree (1) to very strongly disagree (7). The scoring system is to sum scores on all 12 items which gives the total score range of 12-84. Total scores are classified as; 12–48 = Low acuity, 49–68 = moderate acuity and 69–84 = high acuity (Tezel et al. 2011). Ethical approval was granted by the appropriate ethics committee at Trinity College Dublin and permission to conduct this research was granted by the ethics committee at Open Heart House Dublin, Ireland.

Statistical analysis of the data was carried out in SPSS Version 21. The mean, median and standard deviation were summarised for the data and because of the non-normal distribution of the data, non-parametric statistics were applied. Mann–Whitney U and Kruskal–Wallis rank sum tests were used to investigate differences in PSS scores across categorical variables of interest. Reported for each test are the mean ranks for each category. Where Mann–Whitney tests were carried out, the test statistic (U), the Z score and the corresponding *p* value are reported. Where Kruskal–Wallis tests were carried out, the test statistic (H), the degrees of freedom (in brackets) and the corresponding p-value are reported. Questionnaires containing missing responses were not included in this study.

## Results

A total of 75 potential participants (i.e. over 50 years of age), whose names, addresses and telephone contacts were listed on the contact book received an email of invitation and a participant information leaflet from OHH. The response rate for the study was 61 % (n = 46) after excluding questionnaires not fully completed. All 46 (35 males and 11 females) respondents were optimistic about this research and agreed to participate.

## Characteristics of research participants

No statistically significant difference in PSS was observed between the three age categories [*H*(44) = 1.851, *p* = 0.396], although scores were generally lower in the oldest age group (mean rank = 16.25) than in the other two age groups. Age was recorded as a categorical variable on the questionnaire, and hence is analysed as such. The distribution of PSS scores was found to differ significantly for males and females (U = 126.00, Z = -1.714, *p* = 0.044), with males having higher PSS scores in general (mean rank = 24.4) than females (mean rank = 17.45).

**Fig. 1:**
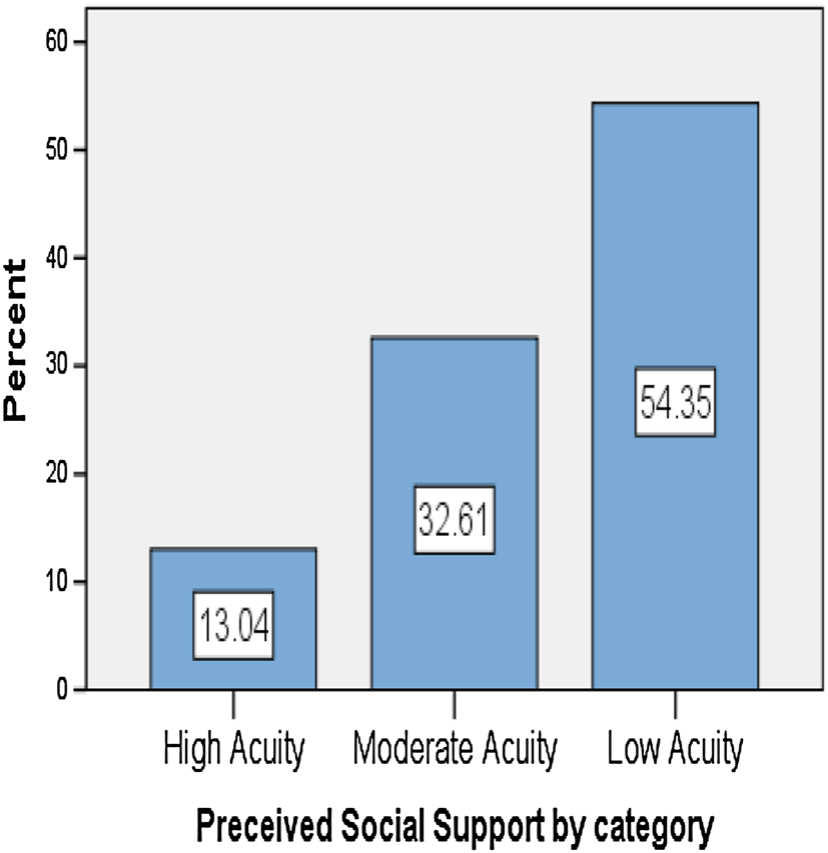
Different levels of Perceived Social Support as a percentage of all the study participants

Figure 1 shows that a little over half of all participants had a low PSS score, fifteen (33 %) had a moderate PSS score and 6 (13 %) had a high PSS score. PSS scores ranged from 23 to 82. The mean PSS score was moderate at 49.13 (SD = 15.823), with a median score of 47 (Tables 1, 2).

**Table 1:** Summary of sample demographics

	Frequency (%)	PSS (mean rank)
*Age*		
50–54	31 (67.4)	23.89
55–59	8 (17.4)	24.63
60-64	6 (13.0)	16.25
Total	45 (100)	
*p* value		*0.396*
*Gender*		
Male	35 (76.1)	25.4
Female	11 (23.9)	17.45
Total	46 (100)	
*p* value		0.044*
*Sexual orientation*		
Heterosexual	30 (67)	23.82
Homosexual	15 (33)	21.37
Total	45 (100)	
*p* value		*0.563*
*Relationship status*		
Single	36 (78.3)	22.32
Married	5 (10.9)	25.3
In a relationship	5 (10.9)	30.2
Total	46 (100)	
*p* value		*0.446*
*Nationality*		
Irish	36 (78.3)	23.06
Non-Irish Caucasian	6 (13.0)	30.08
African	4 (8.7)	17.63
Total	46 (100)	
*p* value		*0.324*
*Duration of HIV infection*		
<2 years	4 (9.1)	16
2–10 years	21 (47.7)	18.14
>10 years	19 (43.2)	28.68
Total	44 (100)	
*p* value		0.020*
*Employment status*		
Employed	9 (20.0)	20.44
Unemployed	26 (57.8)	25.48
Others	10 (22.2)	18.85
Total	45 (100)	
*p* value		*0.322*
*Injection drug use*		
Yes	10 (21.7)	33.55
No	36 (78.3)	20.71
Total	46 (100)	
*p* value		0.003**

Shown are mean rank scores on perceived social support (PSS) and frequency in percent for the categories of all demographic variables

* Statistically significant (0.05 significance level)

** Highly statistically significant (0.01 significance level)

**Table 2:** Summary of sample demographics

	Frequency (%)	PSS mean score (mean rank)	SD	Median	Min–Max scores
*Age*					
50–54	31 (67.4)	50.52 (23.9)	17.42	45	23–82
55–59	8 (17.4)	48.5 (24.63)	6.85	47.5	37–59
60–64	6 (13.0)	42.17 (16.25)	17.1	34.5	28–74
Total	45 (100)				
*p* value	*0.396*				
*Gender*					
Male	35 (76.1)	51.6 (25.4)	16.43	49	27–82
Female	11 (23.9)	41.27 (17.45)	10.96	43	23–54
Total	46 (100)				
*p* value	0.044*				
*Duration of HIV Infection*					
<2 years	4 (9.1)	42.25 (16)	1.5	42	41–44
2–10 years	21 (47.7)	45.29 (18.14)	13.85	43	28–81
>10 years	19 (43.2)	57.47 (28.68)	15.53	59	27–82
Total	44 (100)				
*p* value	0.020*				
*Injection drug use*					
Yes	10 (21.7)	61.7 (33.55)	14.7	65	41–81
No	36 (78.3)	45.64 (20.71)	14.45	43.5	23–82
Total	46 (100)				
*p* value	0.003**				

Also shown are the frequencies, mean, median, standard deviation (SD), minimum (min) and maximum (max) PSS scores for the categories of each demographic variables which were statistically significant

* Statistically significant (0.05 significance level)

** Highly statistically significant (0.01 significance level)

PSS scores were higher in general for those who had HIV/AIDS longer than 10 years (mean rank = 28.68) than for those living with it for <2 years (mean rank = 16.00) and 2–10 years (mean rank = 18.14). A Kruskal–Wallis rank sum test revealed a statistically significant difference in the distribution of PSS scores between the categories of HIV/AIDS duration [H(2) = 7.856, *p* = 0.020]. Individuals who had a history of IDU either past or current had higher PSS scores in general (mean rank = 33.55) than non-IDUs (mean rank = 20.71). A Mann–Whitney U test detected a statistically significant difference in the distribution of PSS scores between IDUs and non-IDUs (*U* = 79.500, Z = −2.678, *p* = 0.006). Reliability coefficient (Cronbach’s alpha) for each of the PSS subscale of special person, family and friends are 0.858, 0.914 and 0.846 respectively. The reliability coefficient of all PSS items is 0.888. Because of the small sample size the validity for this study could not be obtained. Similar population has been studied with proven and acceptable validity in North America (Galvan et al. 2008).

## Discussion

More than half of participants (54 %) were classified as having low perceived social support. These results are supported by similar findings from other literature which found low levels of support (either instrumental or subjective feeling of support) among older adults living with HIV and AIDS. Social support has been consistently associated with the ability to cope with life stressors like HIV/AIDS and other chronic health conditions (Schrimshaw and Siegel 2003; Tezel et al. 2011). Social support provides vital information which help this population age happily, and while longer duration of HIV infection and fewer coping mechanisms lead to low PSS, individuals with certain inherent factors such as being gay and higher resilience have better PSS (Brennan et al. 2011). It is also possible that older adults have fewer social support networks and ways of coping with psychological and social stressors as indicated by their generally lower PSS. This may be because older adults are more likely to lack living parents and/or they may have an older partner with declining health, which may limit both the type and level of support available to them (Chesney et al. 2003; Lyons et al. 2010; Emlet 2006). Conversely, literature suggests that older adults may be more willing to engage themselves in positive relationships and religious activities than younger adults and hence get some kind of external support from other acquaintances (Brennan 2008; Sherr et al. 2009). A statistically significant difference was also observed in the level of PSS between IDU’s and non-IDUs. IDUs reported higher levels of PSS in general than non-IDUs. These findings are supported by similar studies which found lower rates of mental illness and higher social support not only among IDUs in older PLWHA but also in the general population (Whetten et al. 2005; Davis et al. 1995). Substance use on its own is usually sought by individual as a way of coping with elevated level of emotional or social distress, which invariably is associated with low social support (Rabkin et al. 1997). These results may indicate some strong underlying social factors in this population that make IDUs feel less socially isolated and may imply that they have effectively developed a strategy of coping with mental health stressors through peer support, possibly from friends or other IDUs.

This study observed that PSS scores were higher in general among those who had HIV/AIDS longer than 10 years than for those living with it for <2 and 2–10 years. Worthy of note is that the research findings indicate older adults (60 years and above) have been living with HIV the longest compared to those 50–59 years. Firstly, these results could mean that most individuals who were infected during the pre-HAART era no longer view HIV as a death sentence but a manageable chronic illness with the advent of medications (Pitts et al. 2005). Secondly, this result could suggest that they surround themselves mainly with other HIV-positive people and hence are insulated from the social discrimination associated with HIV/AIDS (Cahill and Valadéz 2013; Balderson et al. 2013). Lastly, studies on the aging HIV population need to be interpreted in light of the huge set back stigma pose be it in individual’s willingness to know their status, utilise health care services, join a peer support network or even participate in HIV research, and this must be considered as a potential confounding factor with the ability to skew the research findings so obtained (Heckman et al. 2002). A larger study could be carried out to reinforce the findings presented here.

## Limitations

This study possesses a few limitations. One such limitation is sampling bias, due to- the environment from which the participants were recruited. Open Heart House provides social support to members hence participants in the study sought and received social support through the nature of the organization and may have higher levels of social support compared with others living with HIV. There’s also the possibility of response bias as members of OHH that agreed to participate may be different to those that did not agree to participate. Stigma and resilience which were not included as part of this study are very important variables when it comes to assessing the social support levels of any population and as such should be included in future studies on the PSS of OPLWHA. This study only focused on adults over the age of 50 years without including younger adults, future studies should endeavor to look at these two distinct populations of individual for a more valid comparative analysis.

The small sample size is a limitation and was at least in part due to the inaccessibility of this population and the small population size in general of OPLWHA in Ireland. Lastly due to the small sample size we were unable to confirm the validity of the PSS scale through the use of factor analysis. Factor analysis requires a larger sample size.

## Conclusion

Despite its limitations, this study presents vital information about the low level of social support and injection drug use problems in this population of older adults. Results from this study are particularly important for identifying demographics of those OPLWHA who are most vulnerable to psychological and social distress. An unexpected and yet interesting finding in this study was the positive association between IDU and PSS. Despite the fact that IDU is a feature of mental health problems on its own, results show a greater perceived social support among OPLWHA who had a history of injection drug use, whether current or past history of IDU. Social support in the form of emotional and informational supports provided through the proper channels in this case Open Heart House has been shown to improve the quality of life for older PLHA. However, more can be done by formulating strategies geared towards encouraging older PLWHA to partake more proactively in the community they belong through volunteer services capable of keeping them physically and mentally active, and more intricately involved with the community.

## Abbreviations

ARV: antiretroviral
AIDS: acquired immune deficiency syndrome
HAART: highly active antiretroviral therapy
HIV: human immunodeficiency virus
HSE: health service executive
IDU(s): injection drug user(s)
MSPSS: multi-dimensional scale of perceived social support
OHH: open heart house
PLWHA: people living with HIV and AIDS
SPSS: statistical software for social sciences
SD: standard deviation
